# The alcohol industry, the tobacco industry, and excise taxes in the US 1986–89: new insights from the tobacco documents

**DOI:** 10.1186/s12889-022-13267-w

**Published:** 2022-05-11

**Authors:** Matthew Lesch, Jim McCambridge

**Affiliations:** University of York, York, UK

**Keywords:** Alcohol, Public health, Tobacco documents, United States, Taxation

## Abstract

**Background:**

The UCSF Industry Documents Library has provided public health researchers with key insights into the organization of political activities in the tobacco industry. Much less is known about the alcohol industry. In the US, there is some existing evidence of cooperation between the two industries, particularly in areas where there are mutual interests and/or policy goals at stake. Efforts to raise excise taxes on tobacco and alcohol products are one such example.

**Methods:**

We systematically searched the UCSF Industry Documents Library for data on alcohol industry actors and their political activities. Using content generated by alcohol and tobacco actors, we sought to identify new evidence of collaborations to shape excise tax policy debates in the US in the 1980s and 1990s.

**Results:**

We uncover evidence of the alcohol industry’s efforts to shape excise tax policy debates, both at the national and state level. Excise taxes were defined by both alcohol and tobacco companies and related organisations as a key threat to profits. We show how the alcohol industry confronted this challenge in the late 1980s in the US, uncovering the range of monitoring, coordinating, and public-facing activities used to defeat proposed tax increases at both state and federal levels. The former draws particular attention to Oregon, where alcohol industry actors were not simply operating at the behest of the tobacco industry, but actively led a campaign to advance both brewing and tobacco interests.

**Conclusions:**

The tobacco documents offer a key resource for studying economic interests beyond that of the tobacco industry, operating in collaboration with tobacco companies. Here, brewers advanced shared interests with tobacco, and these findings have implications for advancing understanding of alcohol and tobacco industry political strategies. The findings also suggest that financial documents from other public repositories could be used to generate new inferences about corporate political activities.

**Supplementary Information:**

The online version contains supplementary material available at 10.1186/s12889-022-13267-w.

## Introduction

The UCSF tobacco industry documents archive has been a key source for developing understanding of the internal workings and external influence of tobacco companies [1–3]. A large literature has accumulated, though, in the case of excise taxes, a key policy priority for the industry, there have been only a few studies, with a focus mostly on the United States (US) context given the nature of the available data [4–7]. Across regulatory and policy issues, one common finding has been the importance of coalition-building, in which the tobacco industry has formed alliances with other industries or organisations, in addition, to close working relationships among tobacco companies.

Excise taxes directly affect the profitability of several industries, including tobacco, alcohol, ultra processed food and transportation. During the 1980s and 1990s, as governments looked to pay down deficits or generate additional revenue, excise taxes became a preferred policy lever for many governments. This political environment created new incentives for stakeholder groups, including the tobacco and alcohol industry, to mobilise against these excise tax policy changes [8]. In the 1980s and 1990s, the tobacco industry faced significant reputational challenges [4, 6, 9, 10]. This shift in the political context led key tobacco organisations, including the Tobacco Institute, to play a much less visible role in policy advocacy. According to one study, “the industry sought to create an image of broad support for its [policy] positions in the face of growing public pressure around tobacco control” [6]. Framing policy issues in broader terms can thus be important in mobilising broader coalitions. For example, focusing on the distributive impacts of tax changes, or fairness, has allowed the tobacco industry to build coalitions that include less traditional allies, including progressive groups concerned about the regressive nature of excise taxes [9]. In the case of US federal excise tax policy, tobacco industry actors have previously been found to have recruited representatives from the alcohol and trucking industries, as well as labour unions [9, 10].

Much less is known about the alcohol industry, in comparison with tobacco [11, 12]. It is known that the two industries have cooperated over several policy issues in the US, particularly when their political goals have aligned, including efforts to reduce or minimise rates of taxation [9]. Phillip Morris bought Miller Brewing Company in 1970 and thoroughly controlled how it operated from soon after the purchase. This included influencing public perceptions of alcohol, and promoting alcohol education as a strategy designed to prevent the adoption of policy measures such as raising excise taxes. Attention to alcohol policy was integrated within company-wide thinking, and Phillip Morris was able to play a leading role in key alcohol industry organisations nationally and globally (McCambridge J, Garry J, Kypri K, Hastings G: Using information to shape perception: Tobacco industry documents study of the evolution of Corporate Affairs in the Miller Brewing Company. Globalization & Health, Forthcoming). The company has also been shown to apply marketing strategies developed in tobacco to food through ownership of Kraft General Foods [13].

There are also other longstanding inter-relationships between alcohol and tobacco, so that studies based in the UCSF tobacco industry documents library can yield important insights into the development of long term political strategies by the alcohol industry [14]. The existing literature on the alcohol industry identifies a range of strategies used by actors to influence public policy debates. These give particular attention to framing and lobbying, and also identify a flexible approach to developing novel organisations [15]. Yet understanding of the internal processes that determine the scope of these political activities is limited. The tobacco documents archive presents an opportunity to fill some of that gap, albeit retrospectively given the age of the bulk of the data.

This study seeks to identify the range of activities that alcohol industry actors engaged with in conjunction with tobacco industry actors to advance their common interests vis-à-vis excise tax policy in the US during the 1980s. Excise taxes in the US have been the subject of previous research examining the tobacco documents. Jiang and Liang scrutinized the coordination efforts of tobacco and alcohol companies during the 1980s in the US on excise tobacco taxes, clean indoor air policies and tobacco advertising [9]. They identified the formation of major anti-tax coalitions that functioned at both the national and state level. Our analysis focuses on both sites of political action but does so from the perspective of alcohol industry actors.

Undertaking this study holds potential for better understanding the operations of specific actors within the alcohol industry, including trade associations. The Beer Institute, for example, has evolved into a key coordinating body for the alcohol industry in the US, but has been little studied [16, 17]. Phillip Morris/Miller Brewing Company has recently been shown to have exercised a veto over its activities in 1995, along with the two other major brewers (Anheuser Busch and Coors) (McCambridge J, Garry J, Kypri K, Hastings G: Using information to shape perception: Tobacco industry documents study of the evolution of Corporate Affairs in the Miller Brewing Company. Globalization & Health, Forthcoming.). The analysis can also illuminate specific policy debates. In the 1980s and 1990s, the US federal government and numerous state governments sought out new revenue sources to finance deficit reduction and other expenditures. Exploring how the tobacco and alcohol industry responded to these efforts can offer insights into the organisation of efforts at political influence and the nature of collaborative relationships between the two industries.

The paper is organised as follows. In the next section, we present the methods and data sources used to examine the activities of the alcohol industry. In the Results section we begin by providing some background and context for the detailed analysis by giving particular attention to the Coalition Against Regressive Taxation (CART). The analysis then focuses specifically on the case of Proposition 5 Oregon in which alcohol industry actors led the fight against a ballot initiative to excise raise taxes on beer and tobacco in 1988. This brought together national and state level players in the campaign. We then identify subsequent efforts by the tobacco and alcohol industry to shape excise tax debates at the federal level, specifically the Consumer Tax Alliance (CTA). The study then considers the implications of the findings, taking account of study limitations, and giving attention to issues for further research.

## Methods

We performed searches of the UCSF Industry Documents Library (https://www.industrydocuments.ucsf.edu/tobacco/) between January and March 2021. The first author conducted a preliminary search to assess the availability of materials relevant to the political activities of the alcohol industry in shaping excise tax policy debates. This process was informed by prior experience of examining this resource for alcohol data. Broad search terms were employed, including “taxation”, “excise taxes” along with the names of key alcohol industry actors, including “Miller Brewing Company” and the “Beer Institute.” Based on prior studies [9, 10], we also identified the CTA and the CART as two highly relevant organisations. The first search enabled us to identify specific policy debates that involved both alcohol and tobacco industry actors. The second stage of searches was undertaken in which stricter parameters were applied. The main focus was on documents produced between the 1980s and 1990s and used keyword searches to collect materials on federal and state-level political activities (see appendix). This era was marked by an unusually high degree of public attention to taxation and public revenue in the United States.

Documents were screened, and then analysed, retaining approximately 150 relevant documents. These documents were thematically coded by the first author, and both authors discussed the emerging findings in an iterative manner. The content of documents was cross-referenced with external sources to assess validity, and address information gaps where possible.

Closer examination of state-level activities led us to identify Proposition 5 in Oregon as distinct in that it represented an effort to raise taxes on both alcohol and tobacco, with substantial data available on the roles played by alcohol actors.

## Results

### Context

The Coalition Against Regressive Taxation (CART) operated to shape excise tax policy debates at the federal and state level in the United States, and was formed in March 1986 by business groups – agriculture, manufacturing, transportation, wholesaling and retailing – concerned about the 1986 federal tax legislation. These proposals were eventually abandoned, for which CART claimed credit [18]. CART functioned explicitly as a business lobby group, and in September 1986 claimed that “CART’s efforts attracted enormous public support that caused these proposals to be dropped before they reached the Senate Floor” [19].

Tobacco industry actors, particularly Phillip Morris, saw CART as a key public relations tool, emphasising the “impressive” membership list of CART [20]. The Beer Institute, Distilled Spirits Council of the US (DISCUS), Joseph E. Seagram & Sons, National Beer Wholesalers Association, and Wine and Spirits Wholesalers of America were all involved [18]. All these organisations apart from DISCUS held CART vice president positions [21].

Divisions between different parts of the coalition became evident within CART, and these were reflected within the alcohol industry, for example between beer and spirits, where excise taxes differed [22]. There were questions over whether wine or liquor representatives had been seeking out favourable amendments to the tax legislation that would see their industry spared from higher taxes [23]. A key function of CART meetings seemed to be managing internal conflicts that risked undermining the coalition’s efforts. In 1987, as industry actors were anticipating another tax fight at the federal level, there were reports of divisions with “several CART members, most notably the Beer Institute” beginning to “[pull] out on their own rather than continuing to work under the CART umbrella.” To help “restore solidarity”, the head of the Tobacco Institute, previously recruited from DISCUS, planned to meet with “the beer people” [24]. The Tobacco Institute, the key political actor in the tobacco industry, thus identified managing the unity of the coalition as important to its interests.

CART’s political activities were not restricted to the 1986 tax legislation or the federal level. During the late 1980s, CART also became heavily involved in debates over excise taxes at the state level, particularly as several state governments turned to these taxes as a potential revenue stream. As the next section describes, CART led efforts to defeat a proposed excise tax increase on alcohol and tobacco products in Oregon. According to key financial documents (see below), CART was responsible for administrating the funds used to finance the tobacco and alcohol industry’s campaign against the tax.

### Alcohol industry mobilisation against Proposition 5 Oregon: 1987–8

In the US, several states use initiatives (or referenda) as a way of addressing controversial public policy issues, usually during local, state or federal elections. Initiatives are a specific type of referendum that “[propose] a new law and [reach] the ballot by citizen petition” after the sponsors successfully collect a predetermined number of signatures from the electorate [25]. The policy issues decided through these direct democratic mechanisms have ranged from property tax increases [26] to the definition of marriage [27]. For politicians, one of the main advantages of initiatives is these allow potentially controversial policy decisions to be left in the hands of voters, perhaps explaining why some decisions to raise taxes, including excise taxes on alcohol and tobacco, have been left to initiatives. During the 1980s and 1990s, several efforts got underway to increase excise taxes through voter initiatives. While tobacco was the most common target of these initiatives [28–32], in a couple of instances, alcohol was the subject of a ballot initiative, including Proposition 5 in Oregon (1988) and Proposition 134 in California (1990) [33, 34].

In 1988, in Oregon, voters were asked to approve Proposition 5, proposing to increase existing excise taxes on cigarettes and malt beverages to fund intercollegiate university athletics programs [35]. The main supporters and sponsors of the initiative were the athletic departments of the University of Oregon, Oregon State University and Portland State University [36]. The proposition would increase the tax on malt beverages (e.g., beer, ale) from 6.4 cents to 18.4 cents per gallon but if enacted would prevent the state from imposing any additional tax increases on these alcohol products in the next decade. The proposition would also increase the state tax on cigarettes from 27 to 28 cents per 20 cigarette package. Tax revenue collected from these tax increases was anticipated to be around $8.8 million each year [37].

In November 1987, State Senator Grattan Kerans announced plans to propose the tax increase on the 1988 ballot [38]. In January 1988, proponents filed the initial petition with the Secretary of State, setting the initiative process into motion. Proponents then had seven months to collect the signatures of 63,578 valid petition signers from across the state to qualify for the November ballot [35]. On July 8, proponents of the measure exceeded this threshold submitting over 80,000 signatures [39].

On the November ballot, voters were presented with the following question: “shall taxes on malt beverages (such as beer) and cigarettes be increased in order to finance an Intercollegiate Athletic Fund?” [37]. Efforts largely led by the beer industry were successful in defeating the proposition, with 63% (versus 37%) of voters opposing the tax hike.

### The mobilisation of beer and tobacco actors

Lobbyists representing Oregon’s beer industry were the first actors to mobilise opposition against the initiative. This began shortly after Senator Kerans’s announcement [38]. This primarily took place through CART, which was chaired by Paul Romain [40], an Oregon-based attorney and lobbyist representing the Oregon Beer and Wine Distributors Association [41].

Tobacco industry actors were closely following the tax initiative developments, but had their primary focus on, Proposition 6, which proposed introducing a ban on smoking in most indoor places in Oregon. Tobacco industry actors, including the Tobacco Institute, had retained the services of two Oregon-based consultancy firms Pihas, Schmidt, Westerdahl (PSW) and Public Affairs Counsel (PAC). PSW and PAC had been brought on to assist the industry in defeating the indoor smoking ban [38]. During one meeting, PSW’s President, Mark W. Nelson, updated his clients on the beer industry’s efforts (Nelson had also been conducting work with Coors). This led to some discussion about which firms might be used to help the beer industry mount a campaign against the tax [42]. Given that the tobacco industry was also opposed to Proposition 5, there was keen interest in promoting beer industry activity. PSW recognised the business opportunity and approached Paul Romain. In a proposal written in February 1988, PSW explained:

We have every reason to believe we can defeat the sales tax on malt beverages and cigarettes. That's why we have taken this unusual step of submitting a proposal without being the recipient of a request for proposal [43].

In March 1988, after reviewing several other proposals, CART retained the services of both PSW and PAC [44]. Nelson seemed to function as the main intermediary between the Tobacco Institute and the beer companies. In a March meeting, Nelson advised his tobacco clients that PSW would be the central campaign management and communications firm for the tax campaign [45]. The decision to hire PSW appears to have been made by the beer industry members of CART. In a March 15, 1988 letter, Gary Nateman, head of the Beer Institute, wrote a letter to a Tobacco Institute Vice President to this effect, explaining:

At a coalition meeting of brewers and beer wholesalers held earlier this month in Portland, Oregon to discuss the excise tax initiative, a number of proposals were heard from various campaign consultants. The coalition selected PSW to run the campaign [44].

He also advised the Tobacco Institute that CART would require their financial support to fund the first phase of the campaign’s budget. Nateman requested $40,000 from tobacco companies, as represented by TI. The note also indicated that the beer coalition was regularly meeting to discuss the initiative at G. Heileman Brewing Company and asked the Tobacco Institute to send a representative to the next meeting [44]. Whilst the tobacco industry actors were expected to finance CART’s campaign, the beer producers were leading much of the campaign on Proposition 5.

Part of PSW’s pitch to CART included a detailed vision of its strategy, and specifically how it envisaged the campaign infrastructure being organised. PSW urged CART to create “a steering committee comprised of key industry representatives, their legal counsel and the agency principals” which would have “overall responsibility for coordination of the campaign and consultants” [43].

At the end of March, the industry-led anti-tax initiative campaign began to take shape and followed PSW’s advice. In their first meeting, PSW met with key members of CART, including Paul Romain as well as representatives from Coors, Anheuser-Busch, the Beer Institute, G. Heileman, Miller, Oregon Beer and Wine Distributors, and the Tobacco Institute. These individuals agreed to the campaign’s governance structure at the first meeting, deciding that a senior vice-president of PSW, Ron Schmidt, would be the “single coordinator of all campaign-related information/activities.” The group also decided to create a steering committee that would be “empowered to make final decisions” about the campaign, which would consist of Schmidt, two beer industry representatives and a representative from the Tobacco Institute [46]. The broader campaign committee included five PSW staff and 14 representatives from Coors, Anheuser Busch, the Beer Institute, G. Heileman Brewing Company, Miller Brewing Company, Stroh and the Oregon Beer and Wine Distributors Association. Three individuals from the Tobacco Institute also served on the committee [47]. It is worth noting that several of the committee members were not based in Oregon, and the Oregon Beer and Wine Distributors Association and the Heileman Brewing Company appear to have been the key local actors in addition to PSW and PAC.

### Evidence of alcohol industry leadership

The alcohol industry’s leadership on the initiative campaign was noted in correspondence between tobacco industry actors and seemed to reflect the latter’s preference. In a February 1988 memo to tobacco company executives, George Minshew, a Tobacco Institute Vice President explained to his colleagues:

As you are aware, a second initiative has been proposed in the state of Oregon which calls for excise tax increases on malt beverages and on cigarettes. In all probability malt beverages will take a lead position in developing and conducting a campaign to defeat the proposed tax initiative. *In my opinion the tobacco industry is in its most effective profile working behind the scenes with the malt beverage industry, allowing them to position themselves out-front in this campaign* ([48], emphasis added)

This may have reflected concerns about public perception. One of PSW’s main arguments in its initial proposal was that CART should focus on beer in generating opposition to the tax:

We must recognize the prevailing anti-smoking sentiment and the possibility that audiences might automatically "tune-out" messages which represent smokers. Beer is the commodity which must be used as the focus for building broad based campaign support [43].

In another Minshew letter, additional insight is provided into how the tobacco companies have defined their interests:

In the best interest of the tobacco industry and as directed by the State Activities Policy Committee, we have joined forces with the brewing industry in this initiative development. As approved by the State Activities Policy Committee, we have met with the principals of the Coalition against Regressive Taxation (CART). After approving their direction and campaign procedure, the Tobacco Institute member companies have committed to one-third of the campaign budget, up to a final budget of $800,000 [49].

There is further evidence to suggest that the alcohol industry played a key leadership role in the campaign. According to a presentation delivered to an RJ Reynolds executive in November 1988, the tobacco industry was leading the charge on the indoor smoking ban while the alcohol industry was responsible for “[taking] the lead” on the Oregon tax initiative [50]. This was the agreed division of labour and it suited both sides.

The funding for the Proposition 5 opposition campaign was administered by CART. According to the CART treasurer’s report, this was split as follows; beer producers ($450,000); beer wholesalers and distributors ($150,000); and the Tobacco Institute ($300,000) for financing the $900,000 campaign. Amongst the brewers, this included Anheuser Busch, Pabst, F Heileman, Miller, Coors, and Stroh, with Anheuser Busch making the most significant contribution ($130,000). The four main tobacco contributors were Lorillard, Phillip Morris, Brown & Williamson, and R.J. Reynolds [51]. According to filings submitted to the elections oversight body, CART received $862,140.98 in contributions, with some of the beer producers and distributors, including Pabst, failing to contribute what they had initially committed [52].

### The CART strategy in Oregon

Throughout the campaign, CART used a variety of tactics to oppose the Proposition 5 campaign. Beyond seeking to shape public opinion on the tax, CART also used lobbying and legal challenges. In February 1988, CART wrote to the Secretary of State saying that the proposition violated “the one-subject-only rule.” According to the letter, “proponents of increasing taxes to fund athletics should have to single out the taxing mechanism, and should not be allowed to lump all together to try and use one tax to gather support for another tax” [41]. In March 1988, CART’s chairman, Paul Romain, also filed a petition to the Oregon Supreme Court, arguing that the proposed language failed to “describe the effect of this tax on consumers.” This attempt to appeal the wording of the ballot was unsuccessful [46, 53].

Much of the strategic advice for building a successful campaign was provided by the consultants. PSW proposed an expansive set of state-wide campaign activities, with specific timelines divided between pre-certification and post-certification activities (if proponents collected the required number of signatures). PSW identified several tactics for the former period, including an effort to prevent proponents from gathering sufficient signatures to qualify. This involved developing an “intelligence network regarding proponents' progress in gathering signatures” and initiating “legal challenges and [other] delaying tactics” [54].

CART also gathered information to inform their campaign through multiple sources, including public opinion surveys. A benchmark survey was completed in March 1988 which examined how voters were likely to vote in the autumn election [43]. According to the results, only 42% of voters planned to support the tax, with 52% in opposition and 6% undecided. CART also used the survey to gauge the effectiveness and credibility of certain organisations and individuals, finding that “beer and cigarettes industry representatives [fell] very low on the trust spectrum” while business-oriented organisations had “a higher trust level” [55]. These results came as a surprise to some CART members, particularly the finding that public trust in microbreweries was so low. This led to a subsequent survey designed to clarify the nature of this lack of trust. According to the pollster’s report to CART: “While there [was] some improvement in the rating of the microbreweries they [were] still not high enough to make them a major campaign spokesgroup.” The insights from the benchmark survey and subsequent public opinion polls were also used to develop the content of specific campaign messages as well as identify more politically viable spokespeople (see below) [43]. PSW also advised CART that the campaign needed to build as broad a coalition as possible. This meant engaging with different parts of the alcohol industry who were potentially affected by the tax increase, including smaller producers, wholesalers and distributors, micro-breweries and other relevant trade associations. The consultants were also advised to go beyond the alcohol industry by soliciting the support of organized labour, particularly education unions who would prefer to see funds invested in a different part of the education system. Finally, CART was urged to form alliances with groups whose ideological goals were compatible, including existing anti-sales tax groups as well broader business lobbies such as Associated Oregon Industries (AOI) [46].

Polling, coalition-building, and public relations were seen as mutually reinforcing. Public opinion surveys were used to “develop themelines, slogans and key positioning statements” to inform the content of advertising (see below). Messaging was seen as a key way to broaden the size and nature of the opposition coalition [46]. Between September and November 1988, beer and wine wholesalers distributed campaign material to retail outlets (e.g., taverns, bars and convenience stores) [56]. This underlined the importance of having local distributors as part of CART, allowing the campaign to link up with retailers whose interests were also threatened by the tax increase.

Some of the more expensive elements of the campaign, including advertising and direct mail, were saved for the closing months of the campaign [43]. The bulk of the campaign’s budget was spent on advertising, with $360,000 allocated to television ads, $107,000 to radio ads, $34,000 to print media and $195,000 to direct mail [51]. The messages were carefully tailored based on public opinion research. One of the key themes was the priority that should be attached to the issue; that is, whether funding for an athletics programme was an appropriate priority for the state. This was because, based on their earlier public opinion research, CART knew that the public prioritised other policy issues [57]. In one of their brochures, CART wrote:

[Proposition] 5 does nothing to address the real problems that Oregonians face… problems such as our soaring crime and drug abuse rates, street gangs and underfunded schools, overcrowded prisons and high property taxes [58].

CART also used its direct mail campaign to focus on this theme in not attacking the tax increase directly. The campaign secured the support of the Superintendent of Public Instruction, Verne Duncan. In his letters to voters, Duncan told voters:

Yes, athletics are an important element of higher education, but are they more important than the many other issues currently facing our state? My answer is NO. There are too many other problems we must address first: drug abuse, crime, inadequate funding for education -- to name just a few [59].

CART also used its public opinion research to help inform a much broader range of political messages and issue frames to generate opposition to the proposed tax increase (see Table 1). The use to which the proceeds will be put is a prominent target.

**Table 1:** Additional messages used by proposition 5 opponents

Key Message/Framing	Example
Voters will be left materially worse off from the policy (e.g., loss frame)	“Measure 5 supporters say they want to raise beer and cigarette taxes by nearly $9 million to support intercollegiate sports, but consumers will pay at least $20 million just to raise that amount of tax” “Measure 5 will more than double the state tax on beer. If Measure 5 is approved, more than half of all the state beer taxes collected will go to intercollegiate sports.”
Taxes are a sub-optimal policy option for financing new expenditures	“No college athletic program anywhere in the country is supported or even subsidized by a tax on beer. The other 49 states already know it's a bad idea.”
The anticipated beneficiaries of the new policy are less deserving than the current beneficiaries	“Measure 5 will change the way Oregon has traditionally spent its beer and cigarette tax money. In the past, these taxes have been used to support important human services—such as state, city and county programs to combat mental illness and drug abuse, as well as transportation services for the elderly and the handicapped.”
The policy measure as a slippery slope to higher taxation on alcohol Politicians cannot be trusted not to raise taxes	“Measure 5 supporters say voter approval of their plan will guarantee a 10-year freeze on the beer tax. That's simply not true. Even if Measure 5 passes, the voters, the Legislature or the Federal government can hike the tax again at any time.”

Source: Examples drawn from a campaign leaflet designed for CART by PAC [58]

Proposition 5 was also commonly referred to as “Measure 5” throughout the campaign by proponents, opponents as well as the media

According to the documents, proponents of the tax increase used three main sets of arguments to build public support for the tax. The first claim focused on the nature of the policy problem, specifically that “College athletics in Oregon [had not be] properly funded.” The second category of arguments focused on the competitiveness of athletic programs in the state (e.g., “If the athletic programs at Oregon's colleges and universities had more money they would have more winning teams.” Finally, a third line of messaging stressed the individual health benefits of the policy (e.g., “People shouldn't drink or smoke. Let's tax them. Then they will stop”). According to public opinion research commissioned by CART, only the first argument had much resonance with the electorate [56].

**Fig. 1:**
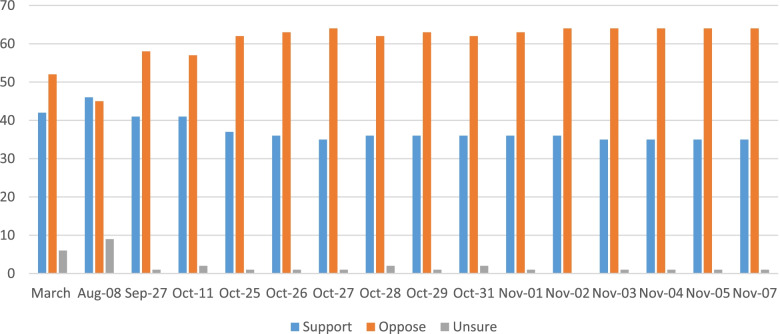
Public support for Proposition 5 in Oregon (1988) Note: This data was aggregated from the UCSF Industry Documents Library (original survey data collected by Public Affairs Counsel)

The impact of CART’s multi-message and multi-media campaign on public support is difficult to assess directly. CART conducted regular polls to gauge public support for the measure. Between the end of September and the November vote, CART commissioned sixteen separate polls. The polls revealed a stable lead for opposition to the tax (see Fig. 1), presumably thereby sustaining the approach being adopted as the ballot approached.

### Industry Anti-Tax Mobilisation at the Federal Level

In 1988, as alcohol and tobacco industry actors anticipated, excise taxes also became a federal issue as legislators looked for a way to pay down the federal deficit. In addition to state-level activities, CART served as the main vehicle for industry opposition during this period at the federal level. Documents show CART engaged in a lobbying campaign in the summer and autumn of 1988 seeking to oppose an increase to excise taxes [60–62]. There are few documents on CART’s activities after this time, as the CTA (see below) became more prominent, though there are indications of financial contributions being carried forward from CART to CTA [63].

The CTA, formed in 1989, was concerned with excise taxes more generally and was designed to serve as “a coalition of public interest groups and labor unions with business support, dedicated to fighting increases in consumer excise taxes” [64, 65]. The Tobacco Institute formed the CTA, and the tobacco companies were the main financial contributors, but this was rarely disclosed [66]. There was often a deliberate effort to emphasise the support and membership of other stakeholders, particularly labour unions [67]. The Tobacco Institute defined excise taxes as a threat to its interests but also recognised “arguing that such taxes hurt sales by reducing smoking was not a viable policy strategy,” The Tobacco Institute saw the regressive nature of excise taxes as a potentially winning argument but believed that the credibility of that claim would hinge on who was advancing the argument [10]. This created incentives for the Tobacco Institute to mobilise a combination of corporate, labour, and populist organisations under the CTA [6, 9, 10].

According to its public-facing materials, the CTA sought “a fairer tax system in which people and corporations who make more, pay more” [67]. Despite claims that CTA membership comprised primarily unions and public interest groups [66], four of the main corporations funding its operations were alcohol companies (Seagrams, Miller Beer, Guinness, and Sazerac). These alcohol producers contributed 12.5% of the CTA’s budget [66, 68, 69]. The CTA’s main aim was to encourage national lawmakers and the general public to look to other more “progressive” policy alternatives for raising revenues and to “remove excise taxes from their list of revenue alternatives” [70]. The CTA formulated several public relations materials as well as wrote letters to legislators arguing that the agreement relied “too heavily on consumer excise taxes” and ignored other “progressive alternatives” [71].

One of the main tactics used by the CTA was a television ad campaign focusing on tax increases for fuel, alcohol and tobacco. The goal of the ad campaign was to “demonstrate that, while an uninformed public consistently supports excise taxes, issue advertising, done properly, can change the public's mind” [71]. Notably, these ads focused on the impact of the tax on alcohol and gasoline rather than tobacco. The CTA conducted pre-polling and post-polling to gauge the effectiveness of the ads after their three-week run. According to one evaluation, CTA had doubled the margin by which the public opposed proposed tax increases [72]. Further polling showed:

the advertising has had a major impact: opposition to consumer excise taxes has increased dramatically in all the markets where the ads were aired [73].

The advertising campaign’s success was then used to garner more financial support for an additional campaign, with the expectation that tobacco companies would contribute $2 million and other participating sectors, including the alcohol industry, would contribute an additional $1 million [66]. The Tobacco Institute emphasised the Beer Institute’s backing for an advertising campaign in their efforts to recruit support from other industries [74], and other key alcohol trade bodies [75].

Despite CTA efforts, congressional leaders reached a bipartisan agreement in October 1990, resulting in the Omnibus Budget Reconciliation Act of 1990. In the case of alcohol, the federal excise tax on beer was doubled, while wine and liquor were also subject to tax increases [76].

The tobacco companies’ efforts to build links with the alcohol industry were not limited to the federal level. The importance of state-level policies on smoking regulations and taxes, meant that there were incentives for them to cooperate at the state level as well. The original Consumer Tax Alliance (distinct from the CTA above) was assembled in California after state-level proposals to raise excise taxes on cigarettes in 1982/83. There were efforts to include the California Beer Wholesalers Association and other alcohol organisations, but these failed to gain support [9].

## Discussion and conclusion

This study draws on evidence from the UCSF tobacco documents archive to better understand the political strategies of the alcohol industry. Focusing specifically on the issue of excise taxes in the US, we add to existing evidence of the alcohol industry’s efforts to shape policy, both at the national and state-level, in collaboration with the tobacco industry. In the 1980s and 1990s, excise taxes were defined by alcohol and tobacco companies as a key threat to both their profits and their positions within policymaking and broader society. Our analysis shows how the alcohol industry confronted this challenge in the late 1980s in the US, specifying the range of monitoring, coordinating, and public-facing activities they engaged in. These policy-influencing activities were pursued in conjunction with the tobacco industry, providing additional evidence of coordination between the two industries identified by previous studies [9] (McCambridge J, Garry J, Kypri K, Hastings G: Using information to shape perception: Tobacco industry documents study of the evolution of Corporate Affairs in the Miller Brewing Company. Globalization & Health, Forthcoming.). Notably, in Oregon, we present an example where alcohol industry actors were not simply operating at the behest of the tobacco industry, but actively led on a campaign affecting both brewing and tobacco interests.

Existing studies have pointed to coordination between tobacco companies and other interests, but the understanding gained from this research, is that the tobacco industry is the pre-eminent actor, manipulating other interests in its own interest [4, 6, 9, 77]. The Oregon case study also provides new insights into how the tobacco industry responded to its increasingly defensive position during the late 1980s. As other studies have documented [10, 31], this era was marked by mounting political pressure for the industry, particularly due to the threat of increased regulation and taxation. Our findings show that the tobacco industry sought to adapt to this shifting political context, aligning itself with other industries which had less unfavourable public images, in this case, the brewing industry. In Oregon tobacco was content to have alcohol lead on Proposition 5, perhaps at least in part, because they were orientated to Proposition 6. Tobacco may or may not have been the more powerful actor in the decision-making, the key point here is that beer was not merely a passive object in the process, the brewers determined that the alliance with tobacco was in their interests, even if it was only in taking their money. The resulting division of labour suited both sets of interests.

The analysis draws particular attention to the range of tactics employed by both tobacco and alcohol industry actors and their allies, showing the political dexterity of these interests. For example, in the case of national-level activities, when legislators withstood this pressure by proposing tax increases, industry actors shifted their focus to the public, seeking to turn public opinion against the idea with their advertising campaigns. When the issue was being debated among politicians, they emphasised the breadth of the anti-tax coalition, continually referencing the membership of CART. When the public began paying more attention, however, CART took a backseat to CTA, which had altogether less the appearance of representing business interests. These findings echo other research findings on the importance of coalition-building activities for alcohol and tobacco companies, flexibilities in forming campaign groups [15] and the use of front groups [4, 6]. Efforts by alcohol and tobacco industry actors to use other campaign groups and actors that have more favourable public reputations has important implications for future study of this subject. It demonstrates the need for researchers to focus their attention on the operation of these issue coalitions, paying particular attention to the membership and funding sources for such groups.

At the state level, our findings provide insights into how the alcohol industry seeks to influence public opinion on excise taxes. In Oregon, the beer industry sought to focus attention on, and minimise the importance of the identified policy problem: a funding shortfall for the state’s post-secondary athletics program. This involved careful targeting. Efforts to raise the excise taxes, then, were presented as a fiscal policy tool. This framing works to distract attention away from other possible reasons for increasing excise taxes on beer and cigarettes, and thus away from the products themselves. This framing, and how it played out in the policy debate, contrasts significantly with more recent efforts to adopt pricing-based alcohol policies such as minimum unit pricing (MUP). In jurisdictions where MUP was adopted, including Scotland, Wales and Ireland, the measure was framed successfully in opposition to industry interests as a measure designed to protect public health [78–82]. The industry’s success in defeating the tax proposal in Oregon suggests that problem-definition and framing are key ideational processes for understanding public receptivity toward alcohol policy measures. The specific nature of the framing tactics of the opposition in Oregon thus warrants careful scrutiny in other contexts, as part of an effort to deepen understanding of the nature of the effects of framing.

The findings also demonstrate the potential value of financial documents, including balance sheets and campaign finance filings, in generating inferences about the alcohol and tobacco industries and their political activities. Polling, political advertising and lobbying required the mobilisation and disbursement of monies for which there are records within the tobacco documents. Such data offer a largely untapped resource for researchers studying corporate political activities alongside the tobacco companies [15]. Financial documents may also exist in other public repositories [83] and thus could be a useful data source in this work.

The methodological challenges involved in working with the tobacco documents archive are well established [1–3, 84]. These documents do not provide a full record of decision-making, and this may be particularly an issue for alcohol actors, whose activities are primarily viewed through the prism of records kept for tobacco purposes. They nevertheless can contextualise and provide insights into some of the key strategic decisions made by alcohol industry actors, with some content more visible than others. The present focus on joint activities of alcohol and tobacco actors has been chosen with these considerations in mind.

A potential limitation of this analysis is that it primarily draws on historical documentary evidence to generate its inferences. The documents analysed here provide a rare window into the private world of corporate decision-making when interests are threatened and political action is needed. There have been important developments over time [85], perhaps most notably with the emergence of so-called social aspects organisations [14], though the consistency in key messages used by industry actors across the decades is striking [14]. Given the under-development of research on the alcohol industry and the scale of the health and social problems caused by its activities, this study makes a valuable contribution to understanding strategic thinking in ways which have contemporary implications. Of course, a broader dataset could offer better context, particularly in understanding the role of other actors such as public officials and the media, but this would not necessarily translate into a deeper understanding of industry actors themselves.

Finally, the findings contribute to broader discussions about the nature and functioning of corporate power and its broader implications for public health [86, 87]. Specifically, the findings help us better understand ways in which corporate actors cooperate across sectors to achieve shared policy goals, including strategies to shape public opinion. The influence of industry actors, particularly in tobacco, alcohol and food, on scientific evidence and public policy is increasingly well established [87, 88]. Researchers have identified several mechanisms that link corporate power to these outcomes but have often lacked data sources to test hypotheses [15, 89]. Closer analysis of documents that already exist in the public domain, including the tobacco documents archive [90], correspondence between industry and government officials [91] as well as campaign finance records [92], provide researchers with data that can help address these gaps.

## Supplementary Information

**Additional file 1**. Appendix. 

## Data Availability

The datasets generated and/or analysed during the current study are available in the UCSF Industry Documents Library repository, https://www.industrydocuments.ucsf.edu/tobacco/.

## Abbreviations

UCSF: University of California, San Francisco
CART: Coalition Against Regressive Taxation
CTA: Consumer Tax Alliance
DISCUS: Distilled Spirits Council of the United States
OBWDA: Oregon Beer and Wine Distributors Association
PAC: Public Affairs Counsel
PSW: Pihas, Schmidt, Westerdahl
US: United States
