# Supplementary material for: The alcohol industry, the tobacco industry, and excise taxes in the US 1986–89: new insights from the tobacco documents
Source: BMC Public Health. 2022 May 11;22:946. doi: 10.1186/s12889-022-13267-w (PMC9097384; doi:10.1186/s12889-022-13267-w)
Supplement: Supplementary file 1 — Additional file 1. Appendix. [file 12889_2022_13267_MOESM1_ESM.docx]

**Appendix**

| **Search strings used in “Truth Tobacco Industry Documents”** | **Hits** |
| --- | --- |
| “Beer Institute” AND “excise tax” | 584 |
| “Consumer Tax Alliance” | 3816 |
| “Coalition Against Regressive Taxation” AND “excise tax” | 2304 |
| “Distilled Spirits Council of the United States” OR “DISCUS” AND “excise tax” | 1338 |
| “Miller Brewing Company” OR “Miller” OR “MBC” AND “excise tax” | 408 |
| “Oregon” AND “proposition 5” | 380 |
| “Oregon Beer and Wine Distributors” AND “tax” | 47 |
| “Anheuser-Busch” OR “Anheuser Busch” AND “tax” AND “Oregon” | 1175 |
|  |  |
| TOTAL | 10,052 |

| **Documents retrieved for analysis** |
| --- |
| 91 relevant documents identified from the search |
| 51 documents cited in the analysis |
